# The Physiological and Pathological Implications of the Formation of Hydrogels, with a Specific Focus on Amyloid Polypeptides

**DOI:** 10.3390/biom7040070

**Published:** 2017-09-22

**Authors:** Létitia Jean, Alex C. Foley, David J. T. Vaux

**Affiliations:** Sir William Dunn School of Pathology, University of Oxford, Oxford OX1 3RE, UK; Letitia.jean@path.ox.ac.uk (L.J.); alex.foley@path.ox.ac.uk (A.C.F.)

**Keywords:** amyloid, hydrogel, physiology, pathology

## Abstract

Hydrogels are water-swollen and viscoelastic three-dimensional cross-linked polymeric network originating from monomer polymerisation. Hydrogel-forming polypeptides are widely found in nature and, at a cellular and organismal level, they provide a wide range of functions for the organism making them. Amyloid structures, arising from polypeptide aggregation, can be damaging or beneficial to different types of organisms. Although the best-known amyloids are those associated with human pathologies, this underlying structure is commonly used by higher eukaryotes to maintain normal cellular activities, and also by microbial communities to promote their survival and growth. Amyloidogenesis occurs by nucleation-dependent polymerisation, which includes several species (monomers, nuclei, oligomers, and fibrils). Oligomers of pathological amyloids are considered the toxic species through cellular membrane perturbation, with the fibrils thought to represent a protective sink for toxic species. However, both functional and disease-associated amyloids use fibril cross-linking to form hydrogels. The properties of amyloid hydrogels can be exploited by organisms to fulfil specific physiological functions. Non-physiological hydrogelation by pathological amyloids may provide additional toxic mechanism(s), outside of membrane toxicity by oligomers, such as physical changes to the intracellular and extracellular environments, with wide-spread consequences for many structural and dynamic processes, and overall effects on cell survival.

## 1. Introduction

Amyloids are proteinaceous aggregates exhibiting a typical cross-β-sheet structure [1,2]. Most polypeptides can form amyloid aggregates under non-physiological conditions [3,4]. Traditionally the term amyloid has been linked with disease leading to the suggestion that non-pathology associated amyloidogenic polypeptides should be regarded as ‘amyloid-like’ or ‘functional amyloid’ [5]. The amyloid fold is considered an ancient structure, characteristic of some of the earliest self-propagating molecules in the prebiotic world [6,7,8]. The amyloid fold has also been proposed to have played a role in the evolution of reversible protein-protein interaction through the formation of zipper motifs. The continued existence and broad distribution of amyloid structures suggest an evolutionarily adaptive function.

In the disease state, amyloid-forming polypeptides must misfold in order to aggregate [5]. The deposition of these misfolded aggregates constitutes the hallmark of many human diseases (e.g., Alzheimer’s disease (AD) and type II diabetes) [5]. The current perception that only disease-associated amyloids are toxic appears incorrect as amyloid-like polypeptides may also form toxic precursors, though these are more tightly regulated to prevent toxicity to the organism [9]. Moreover, disease-causing amyloids are often functional components of larger proteins, which highly regulate the amyloid folding sequences, requiring a conformational or proteolytic trigger to cause aggregation [1,8]. This suggests that diseases result from some imbalance or abnormal circumstances rather than being an intrinsic property of amyloids [1,8]. The evolutionary persistence of the amyloid fold despite its toxicity suggests a functional purpose for amyloidogenesis.

Despite more than 30 unrelated human amyloid-forming polypeptides being disease-associated, there are positive roles for the amyloid fold. These include acting as agents of heritable information (e.g., prions), sequestration of toxic polypeptides, components of biosynthetic pathways, formation of bacterial and fungal filaments, attachment/adhesion functions as well as the formation of biofilms. The genetic and phenotypic variation provided by amyloids underlies the diverse range of functions they fulfil—from facilitating parasitic host invasion (e.g., merozoite surface protein 2 in *Plasmodium falciparum*), reducing competition from closely related species (Microcin E492 produced by *Klebsiella pneumonia*), generating phenotypic diversity by controlling translational termination (Sup35 in *Saccharomyces cerevisiae*), through to protection from freezing in fish, and melanosome biosynthesis (Pmel17 in humans) [10,11,12,13,14,15,16]. This variation also underlies the capacity of infectious amyloids (prions) to act like viruses with distinct strains and host-range specificities, and the ability of amyloids to form some of the strongest tensile materials on earth (e.g., silk in the spider *Nephila clavipes*, chorion proteins (structural components of eggshells) in the silkworm *Bombyx mori*) [17,18]. Hence, the significance of amyloid structures and the breadth of their functions are often under-appreciated.

**Figure 1:**
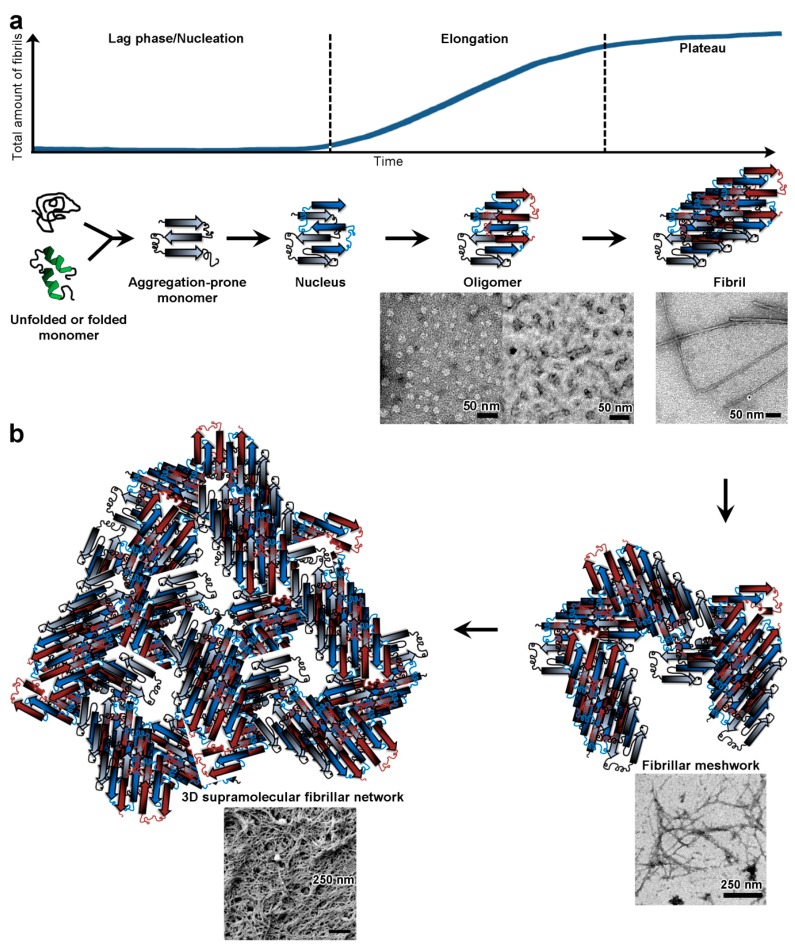
From monomer to hydrogel. (**a**) Formation of amyloid fibrils. Amyloidogenesis is a nucleation-dependent polymerisation process, which shows a typical sigmoidal behaviour. When followed over time, fibril formation can classically be divided into three phases: nucleation typically characterised by a lag phase, elongation and a plateau. During nucleation, monomers (either unfolded or folded) have to undergo a conformational change to adopt an aggregation prone β-sheet conformation. Then aggregation-prone monomers have to come together in the right conformation and orientation, in an energetically unfavourable step, to form the minimal stable assembly, the nucleus. Once formed, the nucleus serves as a structural template for cooperative elongation. The assembly process becomes energetically favourable and proceeds through addition of aggregation-prone monomers onto the nucleus during elongation to form assembly intermediates or oligomers. Morphologically, by transmission electron microscopy, these oligomers appear as spherical structures (doughnut-like of 10–20 nm diameter) or small rods/protofibrils of various length (~20 to 70 nm). Oligomers carry on growing at the expense of monomers until the monomer concentration falls to the critical fibrillar concentration (the minimum monomer concentration required to form fibrils) and then fibril extension ceases (plateau phase). Typically, by transmission electron microscopy, fibrils can be several μm long with a width of 10 to 20 nm; (**b**) Formation of a 3D supramolecular fibrillar network. Beyond fibril formation, amyloid fibrils can interact with one another through a range of non-covalent and non-specific interactions to first form a fibrillar meshwork. By transmission electron microscopy, several μm long fibrils are seen to mostly laterally pack together, as well as twisting around one another. This fibrillar meshwork then proceeds, through further fibrillar interactions and entanglements, to form a 3D supramolecular fibrillar network. By scanning electron microscopy, the 3D network comprises fibril bundles as well as supramolecular networks of condensed amyloid fibrils. In an aqueous environment, this 3D supramolecular fibrillar network would be water-filled and act as the basis for hydrogel formation. This water-filled network has pore size defined by the fibrillar species and cross-linkers if present (see holes in between the schematic entangled β-sheets or within the condensed fibril in the scanning electron microscopy).

Amyloidogenesis is a highly ordered process of nucleated polymerisation (Figure 1a) [19]. First, monomers must adopt a β-sheet structure through conformational changes before forming nuclei, in a thermodynamically limiting step (lag phase). Nuclei then elongate via monomer addition into fibrils. Both amyloid and amyloid-like polypeptides form identical cross-β-folds that result in conserved physico-chemical properties. For example fibrils of Sup35, an amyloid-like polypeptide in *S. cerevisiae,* and of human islet amyloid polypeptide (IAPP), associated with type II diabetes, both form the same class of dry steric zipper [20,21]. Other shared properties include amphiphilicity, adsorption to hydrophobic-hydrophilic interfaces, and once assembled, insolubility, adhesiveness, tensile strength and, for some, hydrogel formation. These features underpin the pathological processes of amyloid diseases but also the processes involved in the biology of many microorganisms.

A hydrogel is a three-dimensional cross-linked polymeric network in which water is the dispersion medium. Despite behaving like a solid, a hydrogel possesses a degree of flexibility very similar to natural tissue due to the high water content within its structure. Many polymers, both naturally occurring and synthetic, fit the definition of hydrogels. Hydrogel-forming natural polymers include proteins (e.g., collagen) and polysaccharides (e.g., starch, chitosan). Although not being universal as some amyloid polypeptides simply precipitate from solution, hydrogel formation has also been reported for a variety of amyloid forming polypeptides, both functional and disease associated [22,23,24,25,26,27,28,29,30,31,32,33].

This review deals with hydrogel formation with a particular focus on amyloid polypeptides in the context of both normal physiology and pathology. Firstly, we describe hydrogels in general, and physical polypeptide hydrogels in term of properties, formation and how their characteristics may be studied. Secondly, we outline the role of natural hydrogels, either cellular or formed by functional amyloid polypeptides, before reviewing that formed by pathological amyloids. Lastly, we link normal physiology to pathology by exploring potential cellular consequences of gelation by pathological amyloids.

## 2. Hydrogels and Physical Polypeptide Hydrogels

As this review deals with hydrogel formation, we are first defining what hydrogels are, along with explaining their characteristics, before describing the types of hydrogels that are the focus of this review, physical polypeptide hydrogels, and how they can be studied.

### 2.1. Hydrogels

Gelation is the phase transition of polymers from liquid phase to gel. Here we do not refer as a gel a solute simply coming out of solution and precipitating. Traditionally, gels are defined as solid and fluid three-dimensional dilute cross-linked systems, which form networks and behave like solids due to the network inhibiting flow at steady-state [34]. Gels can exhibit a range of properties, from being weak to hard, and can expand in any fluid (e.g., water and oil). Gels with water as the dispersion medium are called hydrogels [34,35]. Although over the years, hydrogels have been defined in many ways, one of the common definitions is that water-swollen gels consisting of a cross-linked polymeric network originating from monomer polymerisation that would not dissolve in water, are called hydrogels [34,35,36]. The voids between the polymer chains are filled with water and the polymer network holds the liquid together, while allowing free diffusion of solutes/molecules with various diffusion constants according to their size and shape. Hydrogel density is approximately that of water as they are highly hydrated and adsorbent polymeric networks (the mass fraction of water is greater than that of the polymer). This high water content confers them a high degree of flexibility and viscoelasticity. Macroscopically, hydrogels behave as a solid and microscopically as a liquid.

Hydrogel mechanical strength and physical integrity are provided by cross-linking, either physical or chemical [37]. Physical hydrogels are cross-linked by cooperative, weak and reversible molecular interactions (e.g., hydrogen bonds and/or polymer entanglement), whereas chemical hydrogels use irreversible and stronger covalent bonds. In physical hydrogels, the nature of the cross-linking can create clusters or domains and consequently heterogeneity. Many physical hydrogels become fluid when agitated, but re-solidify when resting (i.e., they can ‘heal’ if they get broken as the non-covalent cross-linking breaks and reforms) [38]. Any changes in cross-linking can alter the hydrogel mechanical properties, e.g., stiffness. Physical gelation implies connections between polymers, through physical mechanisms, to form large scale structures and this can be achieved in different ways: loss of polymer chain flexibility near glass transition temperature (i.e., the atoms or molecular conformations freeze in and arrest molecular motions), loss of molecular mobility for liquid crystalline polymers at their nematic to smectic transition, and polymer aggregation into sample spanning complexes [38].

Preceding hydrogelation, depending on the polymer concentration and solvent composition, liquid-liquid phase separation (LLPS) can occur. During LLPS, a homogenous polypeptide solution separates into a polypeptide-rich phase (also called droplets), formed through weak polypeptide-polypeptide interactions creating a network, and a dilute polypeptide-poor phase [39]. The liquid droplets have a higher density than the solution. With time, droplets grow by stochastically coalescing together until the solution reaches equilibrium. In some cases of LLPS, the droplets percolate the whole system, leading to transition to a gel phase. Recently, a great deal of attention has been given to polypeptide LLPS as this process has been demonstrated to play a crucial role in the formation and maintenance of cellular membrane-less organelles (see Section 3.3). Indeed, studies of fused in sarcoma (FUS) demonstrated that its mutated low complexity region mediates a concentration-dependent LLPS leading to hydrogelation, with the hydrogel composed of polymerised amyloid-like fibrils (see Section 3.3.2) [25,40]. Similarly, it has been shown in vitro that the Phenylalanine-Glycine (FG) repeats of nucleoporins, forming the central channel of nuclear pore complexes, phase transition from liquid to hydrogel via amyloid-like interactions (see Section 3.1.2) [41,42]. Thus, it is clear that a relationship between LLPS and hydrogelation, but also between LLPS and amyloid-like formation, exists. As for pathological amyloids, LLPS has been proposed to explain the hydrogelation heterogeneity of islet amyloid polypeptide, and aggregation into pathological amyloids of low-complexity polyglutamine tracts (as found in huntingtin protein involved in Huntington’s disease) is thought to occur by LLPS [28,43].

Since the first man-made hydrogel (crosslinked hydroxyethyl methacrylate) five decades ago, hydrogels are still of considerable interest due to their current application (e.g., contact lenses) and also their promise in a wide range of applications (e.g., mimicking an environment similar to the extracellular matrix critical in regenerative medicine) [37,44,45,46,47,48]. Indeed, due to their self-supporting viscoelastic and water-filled network, hydrogels allow molecules to diffuse and attach. Biological physical hydrogels (also called natural hydrogels) originates from natural polymers such as polysaccharides and polypeptides. In bioengineering and medicine, and compared to synthetic hydrogels, polypeptide based hydrogels have been favoured due to their biocompatibility and non-immunogenicity [49]. This review focusses on physical natural hydrogels, formed by aggregation, as they are the type of gels formed by amyloid-forming polypeptides.

### 2.2. Physical Polypeptide Hydrogels

Most studies offering detailed characterisation of the process of polypeptide hydrogelation (e.g., conformation, assembly pathway, factors affecting the assembly) have been conducted on synthetic peptides [50,51,52,53]. These peptides are directly relevant for the fields of biomaterials and biomedicine. Although not directly relevant biologically, these peptides behave in a similar manner to full-length amyloid polypeptides in term of conformation and assembly, the main focus of this review, and as mentioned offer the only detailed characterisation of hydrogelation available to date.

Before reaching the gel point, polypeptides are distributed in finite clusters, which are soluble and therefore called a ‘sol’. Beyond the gel point, it is called a ‘gel’. Often physical polypeptide hydrogels are reversible and undergo sol-gel, and sometimes gel-sol, transition. Hydrogel-forming polypeptides generally are amphiphilic [54,55,56] and adopt β-structures (β-sheet, β-turn, hairpin) [57,58,59], but helical structures can also gel [60]. Hydrogel formation by polypeptides is a reversible hierarchical multi-step process during which polypeptide monomers first aggregate by stochastic nucleation before elongating into fibril via monomer addition, with the fibrils then forming a water-filled 3D supramolecular network through fibril entanglement (Figure 1) [61,62,63,64]. Therefore, fibril formation is a necessary step for gelation with fibril density and entanglement determining the rigidity of the hydrogel. Thus, the kinetics of gelation are dependent on the kinetics of fibrillisation. Hydrogels are stabilised by numerous non-covalent interactions, between the polypeptide molecules and/or the polypeptide and solvent [65], such as hydrogen bonds [54,66], hydrophobic [54], ionic [54,57] and π–π interactions, which are typical of amyloidogenesis [58,62]. The properties of the gelled solution are dependent not only on the concentration, length and stiffness of the polypeptide fibrils but also on the number and nature of the crosslinking between them. In particular, we may distinguish fibril entanglement without inter-fibril interactions as one end of a spectrum that extends through global fibril-fibril lateral interactions (e.g., via hydrogen bonding) to specific points of crosslinking determined by associated proteins with bivalent fibril binding properties (e.g., bundling proteins on actin microfilaments—see Section 3.2).

### 2.3. Study of Physical Polypeptide Hydrogels

The properties of hydrogels can be examined using various techniques. Often characterised are the temperatures of sol-gel and gel-sol transitions, by looking at physical state over a range of temperatures, for example by spectroscopic techniques (e.g., dynamic light scattering), thermal techniques (e.g., differential scanning calorimetry) or rheology.

The mechanical properties, and overall macroscopic physical characteristics, of a physical hydrogel can be determined by rheology, which is reviewed by Yan et al. [50]. Rheology permits the study of the relationship between stress and deformation, which can provide polymer characteristics such as gel strength, viscosity (resistance to flow deformation), viscoelasticity (both viscosity and elasticity). The main rheological technique to characterise hydrogels is small amplitude oscillatory shear, during which the sample is subjected to shear flow by small amplitude torsional oscillation. Small strain is preferable as it avoids rupturing the network structure. The rheological measurements in our review were obtained by this technique. The critical gelation concentration can be determined, and the stability, rigidity/strength of the hydrogel can be probed. The temporal evolution of the system can be followed, which allows the sol-gel (and, if happening, gel-sol) transition (cross-over point of the moduli, see below) to be observed. Indeed, the transition strongly affects molecular mobility leading to rheological changes as the solution does not behave as a liquid anymore and not yet as a solid. The linear viscoelastic region of the hydrogel can be determined by doing strain amplitude measurements. Finally, the kinetics of gelation and final gel stiffness can be determined. Generally, three parameters are measured over time, temperature, frequency and strain: the elasticity or stiffness (elastic or storage modulus G’), the liquid-like properties (loss modulus G”) of the solution, and the loss tangent, tan δ (G”/G’). In other words, under shear stress, G’ represents the ability of the deformed polymer to go back to its original state, and G” the tendency of the polymer to flow. As network connectivity increases, both G’ and G” grow, either immediately or after a lag time, before reaching a plateau when maximum connectivity and network arrangement are achieved. Measuring all these changes allow the determination of the gelation kinetics. Typically, for a gel G’ dominates over G” and tan δ < 1, and for a viscous liquid G” dominates over G’. The dominance of G’ over G” over a frequency sweep is also a clear indication of gelation. Importantly, the two moduli have to be independent of the deformation amplitude within the linear viscoelastic region.

Rheological characterisation allows the study of gelation under different environmental conditions and can shed light on the gelation mechanisms. The gelation of many natural polypeptides is not yet fully understood and even less so for amyloid polypeptides, as evident from the very few rheological characterisations given in this review. In the case of pathological amyloid systems, hydrogelation could represent a potential new mechanism for toxicity (see Section 6).

## 3. Cellular Hydrogels

**Figure 2:**
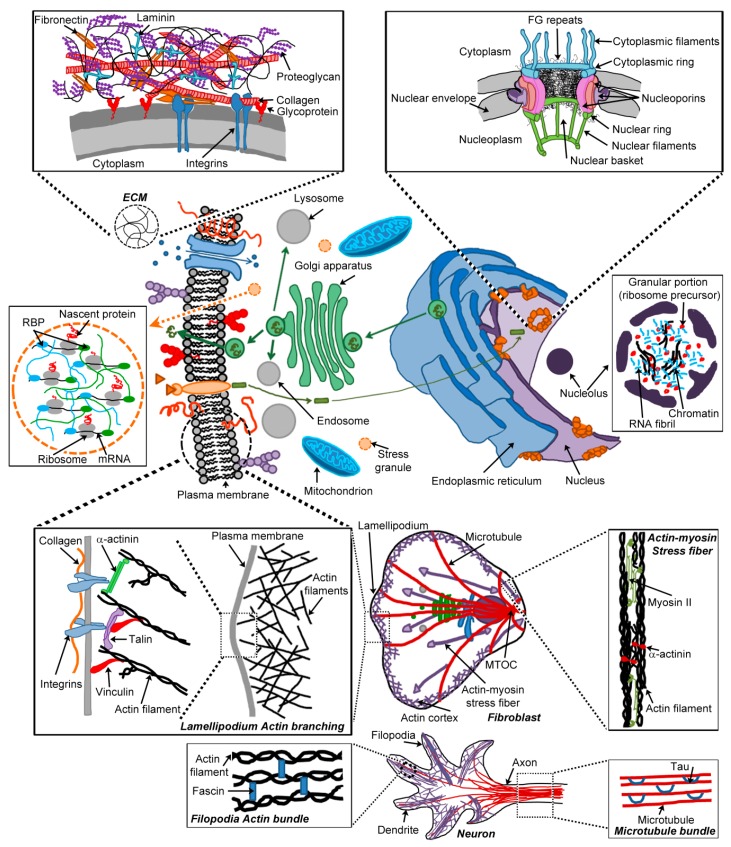
Cellular hydrogels. Hydrogel-forming polypeptides can be found either within the eukaryotic cell (e.g., membrane-less organelles such as the nucleolus and stress granules, the central channel of the nuclear pore complexes (NPC), and the cytoskeleton) or in the extracellular space (extracellular matrix (ECM)). Hydrogel-forming polypeptides provide a wide range of functions for eukaryotic cells: selective diffusion barriers (ECM and NPC), compartmentalisation (nucleolus and stress granules), physical integrity (NPC, ECM and cytoskeleton), and motility (ECM and cytoskeleton). Some cellular hydrogels are formed by ‘functional’ amyloid-forming polypeptides (e.g., the central channel of the NPC and stress granules), but others (nucleolus, ECM and cytoskeleton) derive from non-amyloid polypeptides. For each cellular hydrogel, the hydrogel-forming polypeptide, cross-linkers and any other molecules involved in hydrogelation are depicted. Molecules that are involved in triggering polymerisation and/or polymerisation control, but not in hydrogelation, have been omitted. At the centre of the figure is a schematic of a typical eukaryotic cell, showing organelles and vesicular transport (green circles, containing proteins as green ‘lines’) between organelles of the endomembrane system: endoplasmic reticulum (light blue), Golgi apparatus (green), endosome (grey), lysosome (grey) and plasma membrane. Molecules present at the plasma membrane are also depicted: proteins (red ‘lines’) and glycoproteins (red lines with circles); proteoglycans (purple circles); receptors (light orange), their ligands (dark orange triangles) and downstream effectors (green rectangle); and transmembrane channel (blue) with molecules able to cross through it (blue circles). The ECM (**top left inset**) comprises proteoglycans and fibrous proteins such as collagen (red ‘tubes’), with the precise composition and organisation varying between tissue types. Collagen provides a structural framework for the ECM. Other proteins, such as fibronectin (orange) and laminin (blue), cross-link the ECM itself, but also the ECM to cells (via integrins, blue), and the ECM to soluble molecules. Proteoglycans (black fibrils with purple glycans) form the hydrogel, in which collagen and cross-linkers are embedded. NPC (**top right inset**) are spanning the nuclear envelope and formed from different protein types: filaments and rings (blue and green) forming the cytoplasmic and nucleoplasmic sides, and nucleoporins (purple, orange and pink) spanning the envelope. NPC selectively gate transport between the cytoplasm and nucleoplasm, which is mediated by a subclass of nucleoporins containing multiple Phenylalanine-Glycine (FG) repeats (FG-Nups). FG-Nups form an extended meshwork of fibrils (black filaments) lining the central channel and proposed to form a hydrogel with selective permeability. Stress granules (**middle left inset**) are membrane-less organelles accumulating during translational response to stress. They contain mRNA (black), translation machinery (e.g., ribosomes, grey) and RNA-binding proteins (RBPs; blue and green ovals). RBPs, through their prion-like domains, promote reversible aggregation, liquid-liquid phase separation followed by hydrogelation, which triggers formation of mature stress granules. The nucleolus (**middle right inset**) is also a membrane-less organelle maintained by aggregation, phase separation and hydrogelation. It is organised into three ‘compartments’: the granular portion (ribosome precursors, red), the fibrillar centre (RNA fibrils, blue) and the dense fibrillar portion (chromatin, black). The bottom third of the figure represents two types of cells (fibroblast and neuron), with different types of cytoskeleton organisation detailed (actin, purple, and microtubule, red). Cytoskeleton filaments form hydrogels cross-linked by a range of cytoskeleton-binding proteins. Just beneath the plasma membrane of some resting cells there is a cortex rich in actin. In eukaryotic cells, environment sensing and motility are mostly achieved through two types of protrusions, lamellipodium and filipodia, both formed via actin polymerisation generating treadmilling and driving directional movement at the cell leading edge. In lamellipodium (**bottom left penultimate inset**), actin polymerisation forms a dense network running in a crisscross fashion at angles of ~70°, crosslinked together by filamin (not shown). Directional migration is initiated by extracellular cues such as ECM proteins (e.g., collagen, orange filament). Protrusions are stabilised by adhesions linking the actin cytoskeleton to the underlying ECM proteins. In focal adhesions, integrins (heterodimeric receptor, blue) span the membrane and interact with the ECM substrate and, via actin-binding proteins (α-actinin, green, vinculin, red, and talin, purple), with intracellular actin. Filopodia are long thin protrusions composed of parallel polymerised actin bundles held together by a variety of proteins (e.g., fascin, blue) (**bottom left inset)**. Attachment to the ECM substrate is followed by a contraction phase, detachment at the cell rear and retraction. Retraction requires a motor protein, myosin II (green), found in actomyosin stress fibers, crosslinked by α-actinin (red) (**bottom right penultimate inset**). Activation of the myosin motor leads to shortening of the filaments and subsequent cellular movements, but also promotes disassembly of adhesions at the cell rear. Microtubules radiate from the microtubule organising centre (MTOC) and are involved in moving and redistributing components of the cell. In neurons, reversible microtubule polymerisation in bundle is controlled by tau (blue semi-circle) binding (**bottom right inset**).

Hydrogel-forming polypeptides are widely found in nature and provide a wide range of functions for the organism making them. At a cellular level, hydrogels are found in various locations, from the extracellular space (mucus and the extracellular matrix), to the cytoplasm (cytoskeleton) and within organelles (e.g., the nucleolus within the nucleus, and other membrane-less organelles) (Figure 2). Cellular hydrogels provide various functions, for example selective diffusion barriers, physical integrity and motility. In this section, we do not intend to give a general overview of all cellular hydrogels, but instead we focus on cellular hydrogels that may be targets of non-natural hydrogels formed by pathological amyloids (extracellular matrix and cytoskeleton; see Section 6) and cellular hydrogels made by or linked to ‘functional’ amyloid-forming polypeptides (e.g., the central channel of the nuclear pore complex, secretory granules and membrane-less organelles in the cytoplasm).

### 3.1. Hydrogels as Diffusion Barriers

Diffusion barrier function seems to be a common theme for biological hydrogels. As reviewed by Lieleg and Ribbeck, mucus linings and the extracellular matrix (ECM) are both examples of integral physiological hydrogels that act to selectively filter the exchange of molecules through their polymer matrices [67]. As the review emphasises, the diffusion barrier formed by these gels cannot simply be accounted for via size-exclusion alone. Whilst alterations in ECM porosity in tumour tissue have been demonstrated to affect size exclusion properties, it has also been demonstrated that enzymatic degradation of the proteoglycan decorin or the glycosaminoglycan heparan sulfate disproportionately affects ECM diffusion barrier properties [68,69,70]. This, coupled with the facts that both heparan sulfate and decorin have negative net charges and that the binding of growth factors such as fibroblast growth factor can be modulated by varying the charge density of heparan sulphate side chains, points to electrostatic interactions playing a significant role in the diffusion barrier function of the hydrogel [71,72].

#### 3.1.1. The Extracellular Matrix

In tissues, cells reside within a 3D environment made by the ECM. The ECM is mainly composed of proteoglycans (e.g., hyaluronan) and fibrous proteins (e.g., collagen), with the precise composition and organisation varying between tissue types [73,74,75]. The ECM not only provides physical support for the cells but is also critical for tissue differentiation and growth, and cell signalling. Proteoglycans modulate cell adhesion, migration and proliferation. The most abundant fibrous protein, collagen, provides a structural framework for the ECM, which allows cells to adhere, sense their environment and migrate. Other ECM proteins (e.g., laminin and fibronectin) act as bridges within the ECM itself, but also between the ECM and cells, and between the ECM and soluble molecules. Collagen and fibronectin form a 3D relaxed meshwork of fibrillar structures, which are embedded within a hydrogel formed by proteoglycans. This relaxed meshwork allows both the ECM and the tissue to resist some level of tensile stresses, which implies that the ECM is highly dynamic and undergoes regular remodelling.

Organised layers of ECM, at or near cell surfaces, are also involved in the formation of the basal lamina, which provides physical support for tissues like the epithelium, muscles and the central nervous system [76]. This allows tissue development, differentiation, anchorage and communication between cells within it by protecting it from disruptive stresses. Interactions within the basal lamina ECM are important for intracellular signalling and cell polarity.

ECMs in the brain are rich in proteoglycans and poor in fibrous proteins, and their composition and organisation vary throughout development and in different parts of the brain, with hyaluronic acid being the core organiser [75,77]. Brain ECM plays a vital role in the organ function. In contrast to bulk brain ECM, perineuronal nets are formed by highly organised ECM creating a regular structure surrounding neurons, dendrites and part of axons. Perineuronal net formation is synchronised with the termination of plasticity in the developing brain [77,78]. During development, any perturbation of the ECM around neurons will affect normal growth.

#### 3.1.2. Nuclear Pore Complexes

Nuclear pore complexes (NPCs) fenestrate the nuclear envelopes of eukaryotic cells, and are responsible for selectively gating transport between the cytoplasm and the nucleoplasm. Size filtering alone cannot explain this gating because whilst small molecules (<40 kDa) are able to passively diffuse through the NPC central channel, larger molecules such as mRNA are actively shuttled across [79]. This selective permeability is known to be mediated by a subclass of nucleoporin proteins termed FG-Nups for their multiple repeats of Phenylalanine-Glycine motifs, but the precise means by which they achieve this is still unknown.

The evidence that FG domain-containing Nups are the molecular basis of the NPC’s selectivity barrier is rather robust. Knock out studies have demonstrated that loss of FG domains is either lethal or results in a significant reduction in the permeability barrier in both *S. cerevisiae* and *Xenopus* eggs [40,80,81]. Infection with poliovirus, which proteolytically cleaves three FG domain-containing Nups (Nup98, Nup153, and Nup62), results in disrupted nuclear trafficking and leakage of nuclear proteins into the cytoplasm [82]. Furthermore, FG domains directly bind to nuclear transport receptors (NTRs) [83]. The interactions of FG domain with NTRs are relatively weak [84], but are essential for the translocation of NTRs through the NPC [85,86]. A given NTR is capable of binding to multiple FG-Nups, but will have a strong preference for a small number of the Nups [87]. This is thought to limit the competition for occupancy between NTRs, allowing for multiple NTRs to bind simultaneously. Indeed, there are thought to be roughly 160 NTR binding sites in each NPC, and NPCs have been shown to be capable of facilitating export and import simultaneously [88,89].

The selectivity phase model posits that the identity of the central plug is a homogenous, semi-liquid phase comprising a meshwork of FG-Nups held together by weak interactions between their Phe-rich domains [86]. This is an attractive model with some robust evidence to support it, but it is far from conclusive. These predicted cohesive FG domain interactions have been verified in vitro, and it has proved possible to construct monolayer films of FG domains that mimic the behaviour of an FG domain mesh [90,91]. Finally, and perhaps most convincing, it has been demonstrated in numerous studies that isolated FG domain proteins and derived peptides, both from yeast and vertebrates, can be reconstituted to form hydrogels with selective permeabilities similar to those of the pore in vivo, excluding molecules as small as mCherry (28.8 kDa) [41,92,93,94,95]. However, the presence of a hydrogel lining the central channel of eukaryotic NPCs in vivo has so far not been confirmed.

### 3.2. The Cytoskeleton

Within cells, the cytoskeleton plays major roles, from controlling cell shape and motility to driving chromosome segregation during mitosis. The cytoskeleton comprises microfilaments, microtubules and intermediate filaments. All of these structures form by self-nucleated polymerisation of the following proteins: actin for microfilaments, α- and β-tubulins for microtubules, and diverse proteins (e.g., keratins, desmin and vimentin) for intermediate filaments. Microfilaments are widely distributed throughout the cell, but concentrate, together with myosin filaments, at the cell periphery where actomyosin contractile force generation permits cell crawling and motility. Microtubules radiate from a point within the centre of the cell near the nucleus called the microtubule organising centre (containing the centrosome) and define polarised tracks along which directional motor proteins (kinesins and dynein) move and redistribute organelles. Intermediate filaments span the whole cell in order to strengthen it, but also have positioning and signalling functions. Despite differences with amyloidogenesis, absence of β-sheet structures and highly regulated assembly, cytoskeletal elements also form via nucleated polymerisation giving rise to non-amyloid fibrillar structures which entangle themselves. Indeed, all of these filaments originating from different proteins and locations within the cell assemble in a similar fashion, with protein monomers forming nuclei which then elongate by monomer addition. The filaments then form extended network with rheological properties such as semi-flexibility and viscoelasticity typical of a hydrogel [96]. The local environment, together with associated control proteins that regulate growth and retraction rates, stabilisation and lateral interactions into bundles, confer a dynamic and responsive nature to the cytoskeleton.

Actin is by far the most studied filament within the cytoskeleton. The networks of polymerised actin filaments can be arranged differently within the cell through the action of actin-binding proteins. The cell uses different combination of these proteins to make actin networks with different properties. In microvilli and filopodia, the actin network is arranged in parallel arrays, whereas in lamellipodia, the network is branched with the filament joined at a 70° angle. It is well known that actin filaments form a hydrogel cross-linked by a range of actin-binding proteins (e.g., filamin in lamellipodium, and villin in microvilli) [96,97,98]. Gelation may not depend on the average filament length as long as sufficient cross-linking takes place. However, the binding affinity of the cross-linker and geometry to the filament will also determine whether gelation occurs or not. Indeed, some proteins that cross-link cytoskeletal actin filaments together (e.g., α-actinin) can promote cytoplasmic hydrogel formation, whereas other proteins that cross-link actin in parallel bundles (e.g., villin) do not promote it [96]. Filament stiffness and tensile strength, along with the nucleotide bound (ADP versus ATP), can also influence the gel properties [96].

### 3.3. Membrane-Less Organelles

There is an expanding literature challenging the textbook understanding of how cells spatially create and maintain discrete chemical environments for biochemical reactions, i.e., through partitioning via lipid membranes. We now understand there to be numerous ‘organelles’ that lack these lipid bilayers. These organelles are observed both in the nucleus (e.g., nucleoli, Cajal bodies, and nuclear speckles) and in the cytoplasm (e.g., P granules and stress granules), and vary in size, composition, and function [99,100,101]. Observations of Cajal bodies and *Caenorhabditis elegans* P granules have shown that they behave like liquid droplets [102,103]. Droplets can also be created from reconstituted component proteins of stress granules such as heterogeneous nuclear ribonucleoprotein A1 (hnRNPA1) and FUS [40,104]. Due to their membrane-less organisation, these organelles can rapidly assemble or disassemble in response to cellular needs.

These organelles are remarkable for their ability to sequester proteins and other molecules vital to their functions, whilst maintaining a level of dynamism through incredibly fast turnover of their constituent proteins. All of the membrane-less organelles discovered thus far have contained at least one protein with what has been termed a ‘low-complexity domain’, or a region marked by an overrepresentation of a few residues, and are indicators of a disordered structure. A subset of these proteins contains domains with sequence similarity to yeast prion proteins, predictably termed ‘prion-like’ domains. Also in abundance in many of these organelles are RNA recognition motifs, which make sense given the presence of RNA in most granules and bodies.

#### 3.3.1. Nucleoli

Nucleoli were the first such organelles discovered by early microscopists, but it was only relatively recently that their liquid-like behaviour was demonstrated in *Xenopus* egg nuclei [105]. Nucleoli assemble, after mitosis, onto chromosomal ‘nuclear organiser regions’ that are designated by ribosomal DNA gene repeats and function as ribosome producing ‘factories’ through transcription by RNA polymerase III [106]. Their liquid nature seems appropriate for their function, allowing for the sequestration and rapid turnover of protein machinery and substrates involved in ribosome synthesis. However, the nucleolus is known to have a tripartite organisation, with segregation of protein components involved in different sub-functions into three different compartments, termed the fibrillar centre, the dense fibrillar component, and the granular component, that are identifiable by electron microscopy [107].

How then, can a droplet that behaves like a liquid be organised into three separate components? The answer appears to be that the protein components of the different phases exhibit phase immiscibility and create phases with different surface tensions, embedding one inside the other in a sort of emulsion nesting doll [108]. This discovery adds nuance to our understanding of droplet organelles, suggesting a complexity on par with lipid compartmentalised organelles like mitochondria.

#### 3.3.2. Stress Granules

Stress granules are a specialised type of granule forming and accumulating during translational response to stress. Stress granules form when there is stalled translation due to limiting translation initiation, and comprise sequestered mRNA and RNA-binding proteins (RBPs) [100,109,110]. RBPs contain prion-like domains rich in poly-glycines, which promote reversible aggregation, and formation of mature stress granules involves nucleation by a core primary RBP followed by recruitment of secondary RBPs [111].

Stress granules are an interesting example of droplet organelles, as many of their component proteins have been implicated in pathological states, giving us interesting insights into the nexus of phase separation and toxic fibrillisation. Stress granules are dynamic structures, with the component proteins residing within the granules for anywhere between seconds and minutes, with similar assembly and disassembly times, consistent with LLPS [100]. Indeed, the disease-associated RBPs, hnRNPA1 and the 43 kDa TARDNA-binding protein (TDP-43), have been demonstrated to be capable of LLPS, with hnRNPA1 droplets resulting in fibril formation at high protein concentrations [104]. The rapid formation and disassembly of stress granules (and for that matter, P bodies) is thought to be driven by the prion-like domains of RBPs, as these domains in proteins such as the T cell intracellular antigen 1 (TIA1) are requisite for stress granule formation [112,113]. However, harnessing the rapid assembly properties of prion-like domains does not come without risk. Phase transitions and gelation are likely to increase the propensity for irreversible aggregation. The capacity of hnRNPA1 for fibrillisation at high concentrations is striking given the close relationship between stress granule proteins and disease (see below Section 5.4).

There is clearly a commonality between gelation and LLPS, but the exact nature of the relationship between the two processes is muddled. Both processes involve interactions between low-complexity domains of proteins that draw them out of the phase of their aqueous environment, yet hydrogels and liquid droplets clearly have distinct qualities. The primary difference is the degree of order of the constituent molecules; fluorescence recovery after photobleaching experiments have shown that there is no exchange of proteins in reconstituted FG-Nup hydrogels, demonstrating they are not ‘liquids’ in the sense liquid droplets are [114]. And yet, FUS, in addition to its physiological role in stress granule liquid droplets, has been shown to be able to reversibly form hydrogels or solid aggregates when it is concentrated and subjected to a lowering of temperature, suggesting that these two states may well be on a spectrum of phases these proteins can occupy [25,40]. What is more, stress granule viscosity is not uniform across taxa, with yeast stress granules being near solid gels compared to liquid-like mammalian stress granules, and thought to be a response to the more significant stressful environment yeast cells are subjected to [115]. It further enforces the notion that there might be a wide range of states these proteins can adopt with concomitant changes in biophysical properties and function. To support this, it has been shown that different mRNAs can alter the biophysical properties, including the phase diagrams, of liquid droplets, probably due to differences in binding sites contained within the molecules [64].

### 3.4. Secretory Granules and Peptide Hormones

Secretory cells (e.g., mast cells, neuroendocrine cells, exocrine cells, and peptidergic neurons) all face a common problem: they must find a way of sorting and storing large concentrations of secreted peptides and hormone peptides for extended periods for rapid release upon stimulation. The proteins to be secreted are stored in the dense proteinaceous cores of secretory granules, which also serve as sites for the proteolytic processing and post-translational modification required for peptide hormone maturation [116]. The peptides in these granules are remarkably concentrated, 200 times more concentrated than in the endoplasmic reticulum in the case of prolactin [117].

In elucidating how peptides could be stored stably for such prolonged lengths, it has been demonstrated that the stored peptides form aggregates or crystalline structures within the granules [118]. In a screen of 42 random peptide hormones, 31 were shown to form amyloid aggregates in granule-relevant conditions in vitro [119]. It seems counterintuitive that secretory cells store vast quantities of peptides in the form of amyloid in view of our preconceived notions of amyloids as being toxic and irreversible. However, upon exposure to pH similar to that they would experience upon secretion, all of the aggregated hormones assayed were shown to release monomers. Thus, an efficient environment-dependent reversal of the amyloid formation process is a key intrinsic part of the physiological utility of this type of functional amyloid. The oligomerisation process of these peptides was shown to be moderately toxic, but the authors posit that this effect would be mitigated by the fact the aggregation occurs within the confines of the granule membrane. The ability of these peptide hormones to form amyloid could help to explain how they are sorted into granules as well, as most undergo a self-associated aggregation [120].

Importantly, a quarter of the peptides assayed formed amyloid-like aggregates spontaneously [119]. The others that were shown to aggregate required the presence of glycosaminoglycans, specifically heparan. This makes sense given that proteoglycans and glycosaminoglycans are present in a number of granules in cells, and that an uptick of sulphation and glycosylation of proteoglycans in mouse pituitary tumour AtT20 cells was found upon expression of a secretory protein [121]. The granules of mast cells, which are particularly rich in heparan sulphate were shown by atomic force microscopy to behave like ion-exchange gels [122], teasing the notion that the proteoglycans that assist in packaging peptide hormones and secretory proteins may form a hydrogel within the granules to aid in stabilising the protein aggregates until secretion.

## 4. Natural, Functional and Non-Pathological Hydrogels

A wide range of organisms are exploiting functional amyloidogenesis to provide them with additional functions/properties, and these have been reviewed before [9,14,123,124,125,126]. However, to date, only a few of these functional amyloid-polypeptides have been shown to form hydrogels and these are the ones we focused on in this review.

### 4.1. Microbial Adhesins

Microbial amyloid-adhesins are employed in functions as diverse as forming an adherent growth matrix (e.g., curli with *E. coli*, and *Salmonella* or phenol-soluble modulins with *Staphylococcus aureus*), spore dispersal (fungal hydrophobin), interspecies killing (e.g., Microcin E492 produced by *Klebsiella pneumonia*) and pathogenic microbes interacting with vertebrate hosts (e.g., *E. coli*, *Salmonella*, *Listeria monocytogenes*, *Mycobacterium tuberculosis*, and *Plasmodium* spp.) [10,11,12,127,128,129,130,131,132,133].

#### 4.1.1. Bacterial Biofilms

In addition to the motile, planktonic state, bacteria are also capable of adhering to numerous surfaces they encounter in their aqueous environments, including teeth, industrial pipework, ships, and medical implants, to form sessile biofilms. The formation of these biofilms is a multi-stage process involving loose association between the surface and the bacteria’s glycocalyx, followed by adhesion and the formation of aggregated microcolonies, and finally the development of these colonies into mature biofilms [134,135]. Biofilms are thought to confer numerous benefits on bacterial populations, including providing an environment suitable for complex signalling, differentiation of subpopulations for the division of labour of energetically taxing activities and the pooling of nutrients [136,137]. Furthermore, biofilms are thought to provide protection from a raft of environmental hazards including ultraviolet exposure, desiccation, metal toxicity, and exposure to some antibiotics and antimicrobial agents [138,139,140,141].

The extracellular polymeric substance that enmeshes bacteria in a biofilm has been subjected to rheological measurements demonstrating that biofilms exhibit the classic viscoelastic behaviour of a hydrogel [142,143,144]. The properties of a hydrogel are thought to confer many of the advantages of the sessile state. Most obviously, the viscoelastic properties of a hydrogel provide bacteria within the biofilm with a degree of protection from rapidly changing current conditions they experience in aqueous environments [145]. Moreover, molecules experience a retarded diffusive motion in hydrogels compared to water, allowing for the concentration of nutrients proximal to the cells [67,136]. Beyond entrapping nutrients, the hydrogel is capable of blocking diffusion into the biofilm of other molecules through charge exclusion. Positively charged molecules, such as aminoglycoside antibiotics, show reduced diffusion through biofilms compared to neutral solutes, thought to be a product of the repulsive force produced by the negatively charged biofilm biopolymers, particularly the exopolysaccharides [67,146,147].

#### 4.1.2. Hydrophobins

Hydrophobins are a class of amphiphilic, low molecular weight proteins secreted by filamentous fungi, which have the ability to self-assemble into monolayers at hydrophobic-hydrophilic interfaces [148]. Despite lacking sequence similarity, except for eight cysteine residues that form four disulphide bridges, these proteins form a common β-structured core which is involved in surface adhesion and self-assembly [149,150].

Hydrophobins are separated into two classes. Class I hydrophobins are characterised by the formation of fibrillar ‘rodlets’, which requires a structural rearrangement, and can form membranes that are highly insoluble, requiring harsh acid treatments to be dissociated [151]. The rodlets of SC3 and other class I hydrophobins are considered amyloid-like as they bind Congo Red and thioflavin T, and show an amyloid cross-β sheet structure [152,153,154,155]. The amyloid-like fibrils of SC3 form a semi-permeable protein film with a diffusion cut-off of 200 Da, which, in nature, would allow translocation of amino acids, a few fatty acids and monosaccharides, but not of oligomers of nucleic acids [156]. Class II hydrophobins, on the other hand, do not form such rodlet structures and can be dissociated with either ethanol, cooling, or the application of pressure [157]. The monolayers formed by hydrophobin self-assembly are not strictly speaking hydrogels, but one could imagine that this process represents the first stage in a hydrophobic–hydrophilic interface mediated gelation that subsequently extends into a third dimension, such as with IAPP (see Section 5.1).

The function that hydrophobins fulfil for filamentous fungi is directly related to their surfactant ability; they are some of the most surface-active molecules known [157]. The monolayer formation results in a lowered surface tension allowing the hyphae of fungi in aqueous environments to break through the air-water interface and produce aerial spore-laden conidiophore structures [150]. Moreover, the spores produced are coated in a layer of rodlet filaments, which are thought to convey protection from water by acting as a hydrophobic shield. In some species this layer is further covered in an ECM that facilitates binding of the spores to their host [158].

As with silk proteins (see below Section 4.2), efforts have been made to utilise hydrophobins in drug delivery. Both foams and emulsions of hydrophobins have been used to envelop hydrophobic therapeutic molecules, and it does appear that this coating confers protection from degradation [159]. This is attractive as a means of delivering therapies with slow release, as the hydrophobin coating would break down over longer periods.

### 4.2. Silk

Fibroin light chain (~25 kDa) and heavy chain (~350 kDa), the protein constituent in silk formed by spiders and silkworms, have become an increasingly attractive potential biomaterial and drug delivery system due to their properties. Fibroin is biodegradable over long time periods, is biocompatible, and durable in the extreme, making it highly amenable to biomedical uses [160]. Fibroin is further made attractive as a drug delivery system due to its ability to be easily manipulated into a number of states, including hydrogels [161]. Gelation occurs at concentrations ≤5 wt% and is dependent on a number of factors, including temperature, ionic environment, and pH [162]. Conversion from a liquid to solid fibers occurs through strain-induced phase separation.

Fibroin heavy chain has alternating regions of hydrophobicity and hydrophilicity, with the former comprising numerous repeats of GAGAGS or GAGAGX (X being either V or Y), which can fold into a β-sheet conformation [161]. Detailed rheological characterisations of reconstituted *Bombyx mori* silk fibroin hydrogelation, either at the air-water interface (AWI) or in the whole system (bulk solution and AWI), showed not only formation of viscoelastic interfacial gel-like structures (i.e., films) but also formation of extended gel networks at low concentrations (<2.5 wt% [31,163]. Gelation can be induced by lowering the pH of the solution to around 4, adding Ca^2+^ to potentiate β-sheet formation, or using ultrasonication [162,164,165,166]. These multiple means of creating fibroin hydrogels are key for its prospects as a drug delivery method as it allows molecules with differing sensitivities to be encapsulated within the hydrogel through multiple means without being denatured. Many of the same conditions that determine the propensity of fibroin solutions to gel, such as concentration, temperature, and ion content, also determine the pore size of the resultant gel [162].

## 5. Pathological Amyloid Hydrogels

Amyloidogenesis has been closely associated with an increasing number of diseases including type II diabetes mellitus, AD and Parkinson’s diseases, amyotrophic lateral sclerosis (ALS) and frontotemporal dementia. With ever ageing populations, our understanding of diseases with age-related risk factors, such as amyloid diseases, has become increasingly important. As a result, a great deal of research has been focused upon them over the last few decades. Different amyloidoses are clinically unrelated, but are all characterised by the intra- or extra-cellular deposition of insoluble and misfolded amyloid-forming polypeptides [5]. Instead of precipitating out of solution, some amyloid polypeptides have also been shown to form hydrogels. Outside of islet amyloid polypeptide, TDP-43 and FUS, only partial characterisation of the hydrogel is currently available.

### 5.1. Islet Amyloid Polypeptide

The hallmark of type II diabetes mellitus (in 95% of cases) is the loss of pancreatic β-cells, which is coincident with extracellular deposition of amyloid aggregates formed by a 37 amino acid peptide, IAPP [167]. IAPP and insulin are normally co-expressed by and co-secreted from the pancreatic β cells [168,169]. The loss of insulin control in type II diabetes mellitus is coterminous with the loss of β cell mass. In the insulin resistance state, IAPP amyloids accumulate, which has been shown to be cytotoxic to β-cells [170,171].

Gel formations by IAPP_20–29_ and by full-length human IAPP have first been reported as far back as over two decades ago but it is not until one year ago that hydrogel formation by full-length human IAPP has been characterised [28,172,173]. Jean et al. not only examined hydrogelation in the whole system (bulk solution and AWI, 3D gelation) but also solely at the AWI (interfacial or 2D gelation) [28]. The IAPP 3D hydrogel was shown to contain a three-dimensional supramolecular fibrillar network with classical viscoelastic properties and a storage modulus G’ of 3.6 Pa for a 4 μM solution. IAPP 3D gelation was demonstrated to have complex dynamics with two distinct kinetic regimes, which was postulated to be due to phase separation preceding gelation. The kinetic variation of 3D gelation, and to some extent of fibrillisation, were abolished in D_2_O, suggesting that hydrogen bonding between peptide aggregates, rather than with water, is more important. 3D gelation was also critically accelerated by phospholipids, reinforcing the idea of membranes, and more generally hydrophobic–hydrophilic interfaces, being catalysts of amyloid assembly and thus also hydrogelation. IAPP 2D gelation at the AWI was also demonstrated with a G’ of 6.2 mN/m, a value within the range previously reported for other amyloid hydrogels [128,174,175]. The authors also showed that the onset of fibrillisation and hydrogelation occurred after full IAPP interfacial adsorption, and that hydrogelation occurred concomitantly with fibril extension or fibril extension ceasing.

Another study, by Lakshmanan et al. demonstrated hydrogelation by a 12 mM solution of an hexa-peptide (amyloidogenic and cytotoxic, NFGAIL) derived from human IAPP, with slight phase separation of the fibrillar aggregates from the aqueous phase [33]. Hydrogelation of such a small peptide fragment is not physiologically relevant but shows an association between phase separation and gelation for the IAPP system. The hydrogel contained a three-dimensional fibrillar network, similar to that observed by Jean et al. for full-length human IAPP, and had a storage modulus G’ of ~2000 Pa [28]. G’ for 4 μM full-length human IAPP hydrogel was 3.6 Pa, suggesting a weaker gel for a 3000-fold lower concentration and a 5.4 longer peptide. The IAPP hexa-peptide transitioned to an α-helical intermediate before adopting the typical amyloid β-sheet structure.

### 5.2. Amyotrophic Lateral Sclerosis and Frontotemporal Dementia

There is increasing evidence linking diseases and dysfunction of RBPs. A range of RBPs form fibrillar cytoplasmic and nuclear deposits in neurons that characterise neurodegenerative diseases, such as ALS and frontotemporal dementia (FTD) [176]. Indeed, hnRNPA1 and TDP-43, along with FUS, hnRNPA2B, and TIA1, all have been identified to have mutations that drive inherited forms of neurodegenerative diseases by increasing the aggregation tendency [176,177,178,179,180]. The two most well-characterised proteins associated with ALS and FTD are TDP-43 and FUS, two nuclear proteins that form cytoplasmic inclusions in disease states (though notably these inclusions are exclusive for one or the other protein) [176,181,182]. It has been proposed that formation of these pathological inclusions is driven by the failure of stress granules to disassemble and that pathology-associated mutant proteins interfere with normal stress granule dynamics. TDP-43 and FUS are normally involved in various aspects of mRNA metabolism and stress granule dynamics, and both contain prion-like domains at their C- and N-termini respectively [183]. Mutations within these domains are associated with disease states [177].

TDP-43 has been found to be a major component of ALS and FTD pathological inclusions [184,185]. Both the RNA recognition motif and the C-terminus prion-like domain mediate TDP-43 localisation to stress granules [186]. TDP-43 is not required for stress granule formation, and appears to localise to stress granules in a cell-type and stressor dependent manner [187]. However, TDP-43 does appear to play a role in modulating stress granule formation, as both the speed of assembly and the number of stress granules per cell are affected by TDP-43 knockdown and over-expression [188,189]. ALS-associated mutations in TDP-43 were shown to increase the number and size of stress granules [189,190]. Moreover, the wild-type prion-like domain was demonstrated to reversibly form a hydrogel, which was facilitated by interaction with single strand DNA (ssDNA) [191]. In contrast, interaction with ssDNA of the ALS-associated mutants, A315E, Q331K and M337V, triggered immediate and irreversible precipitation and aggregation, suggesting a loss of the dissociating capability of functional oligomers (i.e., reversibility). TDP-43 contains two highly fibrillogenic sequences within its C-terminus [192]. A tetra-(DLII) and a penta-peptide (NFGAF), from these two fibrillogenic sequences, form fibrillar hydrogels in water driven by hydrophobicity and β-sheet conformation. The hydrogel (formed after 15 days) of 3 mM NFGAF had a storage modulus G’ of 1870 Pa, and that of 2 mM DLII (formed after24 h) had a G’ of 1690 Pa, both comparable to the hydrogel formed by 12 mM IAPP-derived NFGAIL at G’ ~2000 Pa [33].

Similarly to TDP-43, FUS is incorporated into stress granules upon exposure to certain stressors, RNA-binding is also required to mediate ALS mutant FUS toxicity, decreased FUS expression does not abolish stress granule formation, and FUS disease-associated mutations increase the size and number of stress granules [193,194,195]. In contrast to TDP-43, FUS localisation to stress granules only depends on the RNA recognition motif [193]. Mutations within FUS often disrupt the nuclear localisation signal, resulting in cytoplasmic accumulation and increased recruitment to stress granules [181]. Furthermore, ALS/FTD associated FUS mutations within the prion-like domain were shown to trigger liquid-liquid phase separation, and to increase irreversible fibrillar hydrogelation [24,40]. The irreversibility of hydrogelation was due to the mutations since wild-type FUS formed reversible fibrillar hydrogels [24,25,40]. Patel et al. showed that the biophysical properties of the droplets formed by the ALS-associated FUS mutant, G156E, were different to those of wild-type, with slower droplet fusion followed by a complete loss of fusion and eventually to accelerated conversion into fibrils [40]. The N-terminal prion-like domain was shown to be responsible for the capability of FUS to phase transition into a hydrogel [25,40]. The FUS mutant hydrogels were found to have a high viscosity (~15 kPa/s), to entrap other RBPs, to impair local neuronal granule function, and to decrease new RNA translation in axon terminals [24].

### 5.3. α-Synuclein

Amyloid formation by α-synuclein, a presynaptic protein, in Lewy bodies is a characteristic of Parkinson’s disease [196]. α-synuclein, as a native protein, is unfolded but can aggregate into classical cross-β sheet amyloids [197]. The protein can be divided into three regions: the amphipathic α-helical N-terminus when interacting with membrane, the amyloidogenic middle of the protein or the non-amyloid-β peptide (Aβ) component of AD (NAC), and a variable C-terminus [198,199]. Amyloidogenesis of α-synuclein, like that of other amyloid polypeptides, can be catalysed by a secondary nucleation pathway, also known as seeding [200,201]. Seeding requires the presence of preformed aggregates, seeds, which are usually shorter species (e.g., protofibrils or oligomers), which provide conformationally-competent templates for monomer addition [202]. During α-synuclein aggregation, the ‘normal’ nucleation-dependent polymerisation and seeded polymerisation lead to different fibril polymorphisms, ‘straight’ and ‘curly’ respectively [203]. Bhak et al. demonstrated that the ‘curly’ fibrils produced a three-dimensional fibrillar meshwork forming a hydrogel. The hydrogel labelled with thioflavin T, a typical amyloid dye, asserting the amyloid nature of it. The average hydrogel pore size was found to be 52.9 nm by field emission scanning electron microscopy. However, the authors did not characterise the properties of the hydrogel further.

Das et al. used the NAC region of α-synuclein to design hydrogel forming peptides, in which they altered some amino acid side chains to increase hydrophobicity or π-stacking interactions [204]. Hydrogel formation by these peptides was not ‘natural’ but triggered by heating and cooling. The shear thinning behaviour of these hydrogels was studied with rheology, which shows that high strain disrupted the gel network but gel recovery was obtained after strain release. The hydrogels stained with thioflavin T and another amyloid dye, Congo Red, and had a cross-β sheet structure. The authors showed that the gelled fibrils were not seeding fibrillisation of α-synuclein monomers. One of these peptide hydrogels was implanted into adult rat brain, and the implant area showed recruitment of a large number of activated astrocytes and microglia, with the later infiltrating the hydrogel. Although these hydrogels were engineered and formed by small peptide fragments, they were still based on α-synuclein sequence, they reinforce Bhak et al. findings, but importantly this study is the only one to date showing what non-natural hydrogels may trigger in vivo, even if the introduction of the peptide hydrogels into the brain was artificial [203].

### 5.4. Amyloid-β Peptide and Tau

Alzheimer’s disease is the most common cause of dementia. The pathological hallmarks of AD include brain depositions of Aβ, and other proteins, in extracellular senile plaques, and of abnormally phosphorylated tau protein in intracellular neurofibrillary tangles, along with loss of cholinergic neurons in the basal forebrain [205].

#### 5.4.1. Amyloid-β Peptide

The Aβ peptides found in brains of AD patients derived from proteolytic cleavage of a longer precursor, amyloid precursor protein (APP), by β- and γ-secretases. APP is an integral membrane protein found in the synapse of neurons, and the normal function of IAPP and Aβ is unknown. As one of the key player in AD pathology, a great deal of research has been done on Aβ, however, Aβ hydrogelation has been characterised mostly for very small derived peptides.

Only one study showed that full-length human Aβ_1–40_ forms a hydrogel with typical viscoelastic properties and a storage modulus G’ of 10.7 Pa [28]. The remaining experimental evidences for Aβ hydrogelation come from short derived peptides. Although not directly physiologically relevant, the studies on these Aβ fragments shed some light on the properties of hydrogelation (e.g., electrostatic screening, phase separation, 2D conformation of intermediates).

The most amyloidogenic fragment of Aβ comprises residues 25 to 35. While studying the amyloidogenic potential of residues 16 to 20, Krysmann et al. not only showed that this Aβ derived penta-peptide formed typical β-sheet rich fibrils but also a hydrogel [26]. Gelation only occurred in phosphate buffered saline buffer, but not in water, suggesting that screening of electrostatic interactions by the salts is critical. The gel formed had strong viscoelastic properties, with the storage modulus G’ being independent of frequency (i.e., a network had formed), as shown by rheology. In another study, Lakshmanan et al. used hexa- (aliphatic and amyloidogenic sequence from the transmembrane domain, GGVVIA) and hepta-peptides (containing a di-F motif essential for aggregation, KLVFFAE) derived from Aβ_1–42_ and showed that they formed hydrogels at mM concentrations in water, with the fibrils of the hexa-peptide slightly phase separating from the aqueous environment [33]. The hydrogels contained a highly entangled fibrillar network. The hepta-peptide hydrogel was found to be weakly viscous with the lowest G’ value (~200 Pa), and the hexa-peptide had a G’ of ~4000 Pa. These G’ values are much higher than that of 4 μM full-length IAPP (3.6 Pa) and 30 μM Aβ_1–40_ (10.7 Pa), therefore mM concentrations of these Aβ peptides formed stronger and more rigid hydrogels as would be expected for a 3000- and 400-fold difference in concentration, respectively [28]. In contrast to the hexa-peptide, the hepta-peptide did not have an α-helical intermediate or the typical amyloid cross-β structure and formed short and flat β-sheet rich nanotapes rather than nanofibers, with the authors suggesting that this less compact lamellar organisation could explain the low mechanical stiffness of the hydrogel. Lakshmanan et al. also showed that aromatic interactions between the F residues of the hepta-peptide are critical for amyloid nucleation but not for forming a fibrillar network resulting in hydrogelation.

Jacob et al. designed hydrogel-forming di- or tri-peptides based on the β-prone C-terminus of Aβ_1–42_, the most amyloidogenic Aβ species [206]. Only the fluorenylmethyloxycarbonyl (Fmoc) protected peptides formed hydrogels, and the authors showed involvement of intermolecular π–π interactions of the Fmoc. Hydrogel formation by these peptides was not spontaneous but triggered by heating and cooling. Nonetheless, rheological characterisation revealed hydrogel formation with a storage modulus G’ just below 500 Pa (47- and 139-fold higher than that of full-length Aβ_1–40_ and IAPP, respectively), and showed typical self-healing properties [28]. The hydrogels were confirmed to be composed of a dense β-sheet rich fibrillar network, binding both thioflavin T and Congo Red, and shown, as a preassembled gelled mass, not to be toxic to SH-SY5Y cells and to promote cell attachment and mesenchymal stem cell differentiation.

#### 5.4.2. Tau

Tau normal function is in axonal trafficking through microtubule binding and control of their reversible polymerisation. Like hydrogel-forming collagen I of the ECM, tau is very hydrophilic, proline-rich and forms fibrils, suggesting a potential for hydrogel formation.

One study showed that a tau peptide, tau_2–19_, formed a hydrogel at a 6 mM concentration in water after five years incubation, which lacks α-helical structures but is rich in antiparallel β-sheet structures, and requires hydrogen bonding by proline residues [207].

In tauopathies, tau redistributes to, and aggregates in, the somadendritic compartment. Vanderweyde et al. proposed that somadendritic redistribution of tau is linked to stress granule formation [208]. In neuropathologies, stress granules become larger and contain amyloidogenic proteins, e.g., tau [209]. TIA1, a nuclear splicer and a core RBP for stress granule formation, colocalises with brain inclusions associated with a wide range of diseases, from AD to ALS and Creutzfeld-Jakob [111,208,209,210]. Vanderweyde et al. first showed that both TIA1 and tau accumulate concomitantly in brain tissue from rodent models of human tauopathies and in AD human brains [209]. Vanderweyde et al. subsequently showed that tau not only regulates TIA1 interactions with other proteins but also promotes stress granule formation, and that TIA1–tau interaction promotes tau misfolding and aggregation [208]. The authors also showed that the proteins found in the interacting proteome of TIA1 colocalised with phosphorylated tau in brain tissues of tauopathy mice models. Although there is no experimental evidence of hydrogel formation by full-length tau itself, its clear association with stress granules, hydrogel maintained organelles, suggests that pathological amyloids can have dual role in gelation, either being the key gelator or being involved in regulation of gelators.

## 6. Consequences of Gelation by Pathological Amyloids

**Figure 3:**
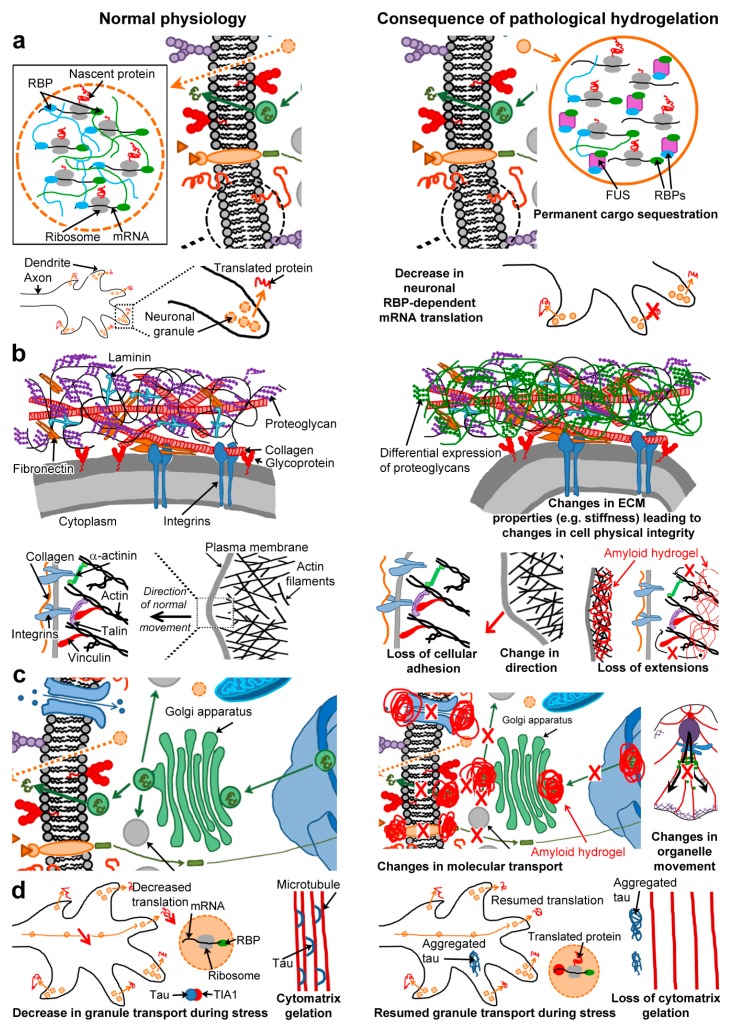
Consequences of hydrogelation by pathological amyloid-forming polypeptides on normal cellular functions. (**a**) Permanent cargo sequestration (**top panels**) and decrease of RNA-binding protein (RBP)-dependent translation of mRNA (**bottom panels**). In normal physiology, cargos are transiently sequestered within granules due to reversible hydrogelation, and are released from them by gel melting (**left top panel**). In neurons, neuronal granules trap, via RBPs, mRNA encoding proteins in close proximity to synapses (**bottom left panel**). In the disease state, pathological amyloid-forming polypeptides, such as fused in sarcoma (FUS), can permanently trap cargo (e.g., RBPs) by forming an irreversible hydrogel (**right** **top panel**). This permanent cargo trapping can affect granule function in several ways. One example would be a decrease in RBP-dependent new translation of mRNA in dendrites and axon terminals (**right bottom panel**); (**b**) Changes in cell physical integrity (**top panels**) and motility (**bottom panels**). In normal physiology, the ECM provides cells with physical integrity (**top left panel**), but also participates in cell motility by linkage with the intracellular actin cytoskeleton (**bottom left panel**). It has been shown that pathological amyloids can affect ECM composition and properties, and can bind to ECM constituents. This would affect ECM hydrogel stiffness and would result in changes in the cell physical integrity by applying mechanical stress (**top right panel**). Disease-triggered changes in ECM hydrogel stiffness would also affect cell motility and migration in different ways (**bottom right panel**): loss of cellular adhesion, changes in rate and direction of cell migration. An inter-relationship between pathological amyloids and the actin cytoskeleton has also been shown. Formation and deposition of additional hydrogels by amyloid polypeptides in the intracellular space may thus alter the actin hydrogel (e.g., flexibility, actin turn-over, actomyosin contractility). This could lead to loss of cellular extensions triggered in different ways: contraction of the actin cytoskeleton (e.g., by increased stiffness), and actin filament depolymerisation (e.g., by a slower diffusion of actin monomers to growing filament ends); (**c**) Changes in molecular transport. In normal physiology, molecules are transported within the cell (vesicular transport within the endomembrane system), outside of the cell (vesicles), or taken up by the cell via receptors or channels (**left panel**). In the disease state, non-natural hydrogels could affect, or even impede, molecular transport in a variety of way: interaction between solutes and fibrils within the hydrogel, reduction of diffusion rates, trapping of molecules bigger than the pore size, and overall by decreasing bulk flow. This would affect the uptake of nutrients and essential molecules, secretion of molecules, intracellular trafficking, vesicle content might be released in the wrong place, movement of signalling molecules between cells, and movement of organelles (**right panels**). Sites where native hydrogels are absent or have very different properties would be the most affected; (**d**) Regulation of non-pathological gelators. In neuron normal physiology, translation of mRNAs relies on RBP-dependent RNA transport in neuronal granules to the synapse (**left panel**). Tau was proposed to slow RNA granule transport to the synapse, and therefore to be involved in translational stress response, due to its interaction with RBP T cell intracellular antigen 1 (TIA1). Tau, as a microtubule regulator, could also play a role in the cytomatrix hydrogelation. Thus, amyloids like tau, when in a monomeric state, can be involved in regulation of non-pathological gelators. In the disease state, as soon as amyloid-forming polypeptides start aggregating and gelling, their role in regulating hydrogelation of other components might be affected or abolished (**right panel**).

The absence of cytotoxicity in organisms using the amyloid fold to fulfil physiological functions suggests some level of regulation (proteostasis to regulate abundance, gatekeeper residues to limit assembly to certain circumstances). Fibrillisation is also used as a mechanism for storing peptide hormones in *Homo sapiens* (see Section 3.4) [119]. However, fibril fragmentation is also a general property of amyloidogenesis, which is useful in the release of peptide hormones but more problematic in regards to toxic intermediates. In normal circumstances (i.e., in absence of a trigger), fibrillisation may simply act as a sink for toxic species, to allow cellular machinery to process the compound at a rate which doesn’t interfere with cellular function. Fibrils themselves seem not to be causal for disease (e.g., the poor correlation between AD clinical severity and amyloid deposits) and are viewed as stable non-toxic proteinaceous structures [211,212]. The current view is that oligomers or intermediates of the assembly pathways are the toxic species causing disruption of membrane integrity in various ways (e.g., carpeting and pore formation) [213]. Here, we propose that, additionally, non-natural hydrogel formation, as triggered by some pathological amyloids, could also physically change both the intracellular and extracellular environments of cells, affecting/disrupting all sorts of processes, from motility to molecular transport, and overall cell survival (Figure 3).

### 6.1. Permanent Cargo Sequestration (Figure 3a)

In normal physiology, formation of reversible hydrogels transiently sequesters cargos, which are released upon gel melting (see Section 3.3.2). However, in the disease state, permanent trapping of cargos by irreversible hydrogelation could impede a range of processes. The properties of irreversible hydrogels are emergent and depend not only on the characteristics of individual fibrils (length, stiffness, branching, twist, termination vs. continuous growth) but also on the characteristics of the intersection points, whether they are lateral hydrogen bonds (global dependence on pH and ionic strength) or specific crosslinking proteins in the case of the cytoskeleton (especially actin). Cargo trapping by irreversible hydrogelation was shown for ALS/TDP associated mutants of FUS, which permanently trap other RBPs and affect ribonucleoprotein granule function (see Section 5.2) [24]. In the brain, mRNA encoding synaptic proteins are localised within neuronal granules, through the binding to RBPs, where they can orchestrate mRNA translation in close proximity to synapses [214]. Therefore, perturbation of cytoplasmic ribonucleoprotein granule function can in turn decrease RBP-dependent new translation of mRNA in dendrites and axon terminals, leading to neuronal malfunction.

### 6.2. Cell Physical Integrity and Motility (Figure 3b)

Tissue stiffening can arise due to inappropriate collagen level or crosslinking, which compromises the ECM normal function and therefore affects cell functions, as seen in aging and cancer [215,216]. ECM stiffening can result from increase deposition of large quantities of ECM proteins by locally recruited fibroblasts during acute injury [217]. Stiffer ECM not only puts mechanical stress on cells but also can lead to loss of cellular adhesion and of apical-basal polarity by disrupting the basement membrane. In amyloid pathologies like AD, it has been shown that the ECM proteoglycans are differentially expressed in a way that may inhibit neuronal repair [218]. Moreover, in familial amyloid polyneuropathy extracellular deposition of transthyretin in the peripheral nervous system was shown to be accompanied by changes in proteoglycan type and distribution, and upregulation of matrix metalloproteinase-9 (involved in degrading ECM components) leading to tissue remodelling [219]. Similarly, Aβ, one of the key players in AD, has also been shown to upregulate matrix metalloproteinase-2 and -9 [220]. Thus, it is clear that pathological amyloids can affect ECM composition and properties.

In order to move within a tissue, cells migrate through the ECM hydrogel. This cellular movement depends on the hydrogel properties (e.g., stiffness), whether the hydrogel constituents can be rearranged (e.g., local proteolysis or melting), adhesion strength to the hydrogel, and how strongly the cell can propel itself [221]. Therefore, local differences within the ECM hydrogel will influence the rate and direction of cell migration, even in the presence of a chemoattractant gradient. Cells use plasma membrane protrusions propelled by networks of polymerised actin in order to attach to the ECM and migrate through it, but also use contraction provided by actin-myosin stress fibres to remodel their immediate surrounding. Thus, the cell uses an intracellular gelled network in order to move through an extracellular gelled network (see Section 3.1.2  and Section 3.2). However, formation of additional hydrogels by some amyloid polypeptides and their irreversible deposition in the intra- or extracellular space may affect cell movement. Indeed, extracellular deposition may affect the properties of the ECM hydrogel (e.g., pore size, stiffness) and intracellular deposition may affect the cytoskeleton hydrogel (e.g., flexibility, actin turn-over, actomyosin contractility).

With regards to the ECM, it has been shown that in restrictive cardiomyopathies, amyloid deposition occurs in the ECM and results in an increase in myocardial ECM volume, which in turn causes myocardial wall thickening [222]. Furthermore, transthyretin amyloid build up was also shown to alter myocardial ECM stiffness in cases of transthyretin cardiac amyloidosis [223,224]. APP, the precursor of the AD Aβ, possesses a heparin-binding domain and was shown to interact with collagen and to promote cell–cell and cell–substrate adhesion [225]. These properties would of course be completely altered as APP is proteolytically cleaved to produce Aβ, as well as subsequently when Aβ assembles into a fibrillar hydrogel network, which in turn would affect neuronal movement. Similarly, serum amyloid P component was also shown to bind selectively to ECM components (e.g., fibronectin) [226]. Therefore, the ability of some pathological amyloids to bind to ECM constituents suggests that pathological hydrogel formation can occur very close to or even within the ECM itself, which would affect its composition and properties. ECM components have been shown to associate with or accumulate in neuronal amyloid plaques during AD (e.g., laminin, proteoglycans such as heparin sulfate), again reinforcing the idea of a close relationship between amyloids and the ECM [227].

The ECM, as a passive molecular sieve, also affects intracellular processes. Cells can respond to changes within the ECM hydrogel (e.g., stiffness or structure) by changing their cytoskeleton, motility and proliferation, with different cell types reacting in different ways [228,229,230,231]. For example, neuronal stem cells proliferate less when the ECM hydrogel becomes stiffer [232]. Any changes of the ECM hydrogel properties would affect cell access to essential molecules (e.g., nutrients and oxygen), which would significantly influence cellular activities, but also cellular movement.

In neurons, actin filaments are abundant in growth cones and dendritic spines, which play a crucial role in neuronal plasticity [233]. Any changes in actin polymerisation in dendritic spines would then alter plasticity and this has been associated with neuropathologies such as AD [234]. Based on the crucial role of actin in synapses (formation and maintenance), any changes in actin polymerisation or its control would lead to synaptic dysfunction. There are many lines of evidence showing an inter-relationship between amyloid-forming polypeptides and the cytoskeleton and below are some examples.

The Finnish type of familial amyloidosis, an autosomal dominant form of systemic amyloidosis, is characterised by amyloid aggregation and deposition of gelsolin, an actin-severing and capping protein, resulting in the loss of actin-binding capability [235,236]. In the case of AD, Heredia et al. showed that hippocampal neurons treated with fibrillar Aβ have increased levels of inactive Ser3 phosphorylated ADF/cofilin (an ADP-actin severing protein) and active Thr508 phosphorylated LIM kinase 1 (an ADF/cofilin inhibitor by phosphorylating Ser3), resulting in dramatic remodelling of actin filaments (actin filament accumulation) and neuronal degeneration [237]. They also showed that, in AD brains, the number of LIM kinase 1 positive neurons is increased in areas affected by the pathology, and that these neurons also contain intracellular Aβ and pre-tangle phosphorylated tau. This and other studies showed that Aβ deposition was linked to an increase in actin aggregation and polymerisation, and that polymerised actin aggregates are observed in hippocampal sections of AD brains but not in those of controls [237,238,239]. Song et al. demonstrated that Aβ can also induce actin stress fibers in a septal neuronal cell line [240].

In AD, dendritic retraction occurs concomitantly with neurofibrillary tangle appearance, which suggests that the cytoskeleton properties must be altered and that tau might play a role. Tau_2–19_ was shown to form a hydrogel (see Section 5.4.2). Tau, through its normal function in regulating microtubule polymerisation, is associated with the cytoskeleton. Thus, one could hypothesise that hyperphosphorylated tau aggregation would be embedded within the cytoskeleton meshwork, leading to tau hydrogelation (if it does occur for full-length tau, as hydrogel formation as only been shown for tau_2–19_) being enmeshed with that of the cytoskeleton. Tau hydrogelation would then modify or perturb an existing cytoskeleton hydrogel or alter the formation of a new one. If this perturbation leads to depolymerisation (e.g., by a slower diffusion of actin monomers to growing filament ends) or contraction of the cytoskeleton (e.g., by increased stiffness), cellular extensions such as dendrites would not be supported but in addition focal adhesions with the ECM would be destabilised.

There are many more lines of evidence of the inter-relationship between pathological amyloids and the cytoskeleton, and to fully detail all would be beyond the scope of this review. However, it is highly likely that the cytoskeleton polymerisation is a target of pathological hydrogel formation, which would not only perturb cell motility but also vesicular transport.

### 6.3. Molecular Transport (Figure 3c)

Any molecular transport could be hindered by non-natural hydrogels for a variety of reasons; a possible interaction between solutes and the fibrils within the hydrogel, a reduction of diffusion rates, the trapping of molecules bigger than the pore size, and overall by decreasing bulk flow through hydrodynamic drag and steric hindrance. As shown by Woodard et al., diffusion of ions and molecules smaller than the hydrogel pore size, would only be insignificantly reduced (1.2% reduction for a 2% fibril volume fraction of a 20 mg/mL lysozyme hydrogel) [241]. Thus, diffusion would only be marginally affected by non-natural hydrogels. Therefore, movement of molecules within the aqueous phase of a hydrogel would occur through the fibrillar meshwork whenever there is bulk flow of solvent and pores of a certain size (>100 nm) [242,243]. However, such pore size has been shown not to be stable and to rapidly shrink into smaller sizes due to the hydrogel expanding into them [242]. Moreover, the effective pore size will depend on the surface charge on the hydrogel and the charge properties of the solute, giving rise to selective differences in diffusion for specific solute classes. Exclusion of positively charged molecules such as aminoglycoside antibiotics from bacterial biofilm hydrogels with negative surface charge is a good example of this effect (See Section 4.1.1).

The outcome of this combination of physico-chemical effects of non-natural hydrogels will be to selectively impede molecular movement/transport. The consequences would be most serious at sites where native hydrogels are absent or have very different properties. This would affect not only the uptake of nutrients and essential molecules, but also excretion of waste and secretion of other molecules. This would be critical in organs/tissues that are not normally rich in hydrogels, such as the brain, but especially for organs like the brain or the pancreas, which rely heavily on secretion to perform their functions; i.e., secretion of neurotransmitters by neurons and of insulin by β-cells respectively. The movement of signalling molecules between cells would also be hindered by local extracellular deposition of non-natural hydrogels, which again would be critical in the brain with decrease/impairment of movement of neurotransmitters in the synaptic space.

Local intracellular deposition of non-natural hydrogels could also impede vesicular transports and movement of organelles, as it was shown for hydrogel formed by the cytoskeleton [244]. Active vesicular transport along microtubules, e.g., between endomembrane organelles or axonal neurotransmitter transport, would be hindered or blocked whenever vesicles would be larger (40–100 nm) than some of the hydrogel pores or when transporting microtubules would be embedded within the hydrogel. Consequently, not only trafficking would be impeded, e.g., reduction in synaptic release, but also vesicle content might end up being released in the wrong place, e.g., at the soma or on dendrites rather than at the synapse. In several neurodegenerative diseases, including AD, axonal transport has been shown to be impaired, with greater synapse loss than neurons in early stages of AD [245]. Similarly, axonal transport has been shown to be damaged in rodent models of ALS, with retrograde axonal transport impediment occurring in presymptomatic mice [245].

### 6.4. Regulation of Non-Pathological Gelators (Figure 3d)

In neurons, translation of mRNAs into proteins is dependent on synaptic activity, with RNA transport to the synapse being dependent on RBPs. During stress, tau redistributes to and aggregates in somadendritic compartments, which has been proposed to be linked to stress granules formation [208]. Due to tau interaction with the RBP TIA1 and tau involvement in regulating TIA1 interaction with its binding partners during stress granule formation, tau was proposed to slow RNA granule transport to the synapse and therefore to be involved in translational stress response. Stress granules are membrane-less organelles maintained through aggregation and hydrogelation of some of their components as part of normal physiology (see Section 3.3). Similarly, the cellular cytomatrix is a dynamic hydrogel formed from microtubules, actin filaments and intermediate filaments, which is triggered or maintained by a range of other proteins (see Section 3.2). Due to its microtubule assembly function, tau could also play a role in the cytomatrix hydrogelation. Thus, pathological amyloids like tau, instead of being key gelators themselves can be involved in regulation of non-pathological gelators.

In the case of tau, its involvement appears to be ‘beneficial’ for stress granule formation. However, one could imagine that an amyloid-forming polypeptide could be involved in normal physiology when in a monomeric state, but that as soon as it starts aggregating and gelling its role in regulating hydrogelation of other components might be affected or abolished.

## 7. Conclusions

Polypeptides polymerising into a water-swollen and viscoelastic three-dimensional cross-linked polymeric network, called a hydrogel, are widely found in nature. Hydrogels provide a wide range of functions for the organism making them, either at the organism or cell level.

Cross-β sheet amyloid structures can be damaging or beneficial to different types of organisms. The best-known amyloids are those associated with diseases (e.g., Alzheimer’s and Parkinson’s diseases, and type II diabetes mellitus). However, a wide range of higher eukaryotes and prokaryotes utilise the amyloid fold to maintain normal cellular activities or to promote survival and growth. Both functional and disease-associated amyloids use fibril cross-linking to form hydrogels, which can be exploited by organisms to fulfil specific physiological functions.

Outside of the well-studied nucleation-dependent polymerisation, investigation of hydrogelation by pathological amyloids is an emerging field and characterisation of their hydrogel properties is lagging behind that of functional amyloid hydrogels.

Cellular malfunction and death in amyloid diseases may not only be due to oligomer toxicity via membrane perturbation, but also due to the physical effects of the formation of non-natural amyloid hydrogels on cellular functions. Insights into the mechanistic role played by hydrogels in amyloid diseases may teach us more about native hydrogel functions to develop new polypeptide-based hydrogels for use in bioengineering and medicine, but the major prize will be a better understanding of disease progression leading to novel future treatments.

## Abbreviations

air-water interface	AWI
Alzheimer’s disease	AD
amyloid-β peptide	Aβ
amyloid precursor protein	APP
amyotrophic lateral sclerosis	ALS
extracellular matrix	ECM
frontotemporal dementia	FTD
fused in sarcoma	FUS
heterogeneous nuclear ribonucleoprotein A1	hnRNPA1
human immunodeficiency virus	HIV
islet amyloid polypeptide	IAPP
liquid-liquid phase separation	LLPS
microtubule organising centre	MTOC
non-amyloid-β component of AD	NAC
nuclear pore complex	NPC
nuclear transport receptor	NTR
RNA-binding protein	RBP
T cell intracellular antigen 1	TIA1
43-kD TARDNA–binding protein	TDP-43
